# 

**Published:** 1941

**Authors:** 


### CONTENTS.

| | Page |
|---|---|
| **Thyrotoxicosis.** By A. Rendle Short, M.D., B.S., B.Sc., F.R.C.S. | 1 |
| **The Importance of the First Year of War in Mental Disease.** By R. E. Hemphill, M.D., D.P.M. | 11 |
| **Sex Hormones and Blood Pressure.** By A. Guirdham, D.M., B.Sc., D.P.M. | 19 |
| **Reviews of Books** | 22 |
| **Editorial Notes** | 27 |
| **Obituary:** W. H. C. Newnham, M.A., M.B., M.R.C.S. | 31 |
| **The Medical Library of the University of Bristol** | 32 |
| **Publications Received** | 35 |
| **Local Medical Notes** | 36 |

